# Supplementary figures and images for: Schistosoma haematobium Extracellular Vesicle Proteins Confer Protection in a Heterologous Model of Schistosomiasis
Source: Vaccines (Basel). 2020 Jul 24;8(3):416. doi: 10.3390/vaccines8030416 (PMC7563238; doi:10.3390/vaccines8030416)

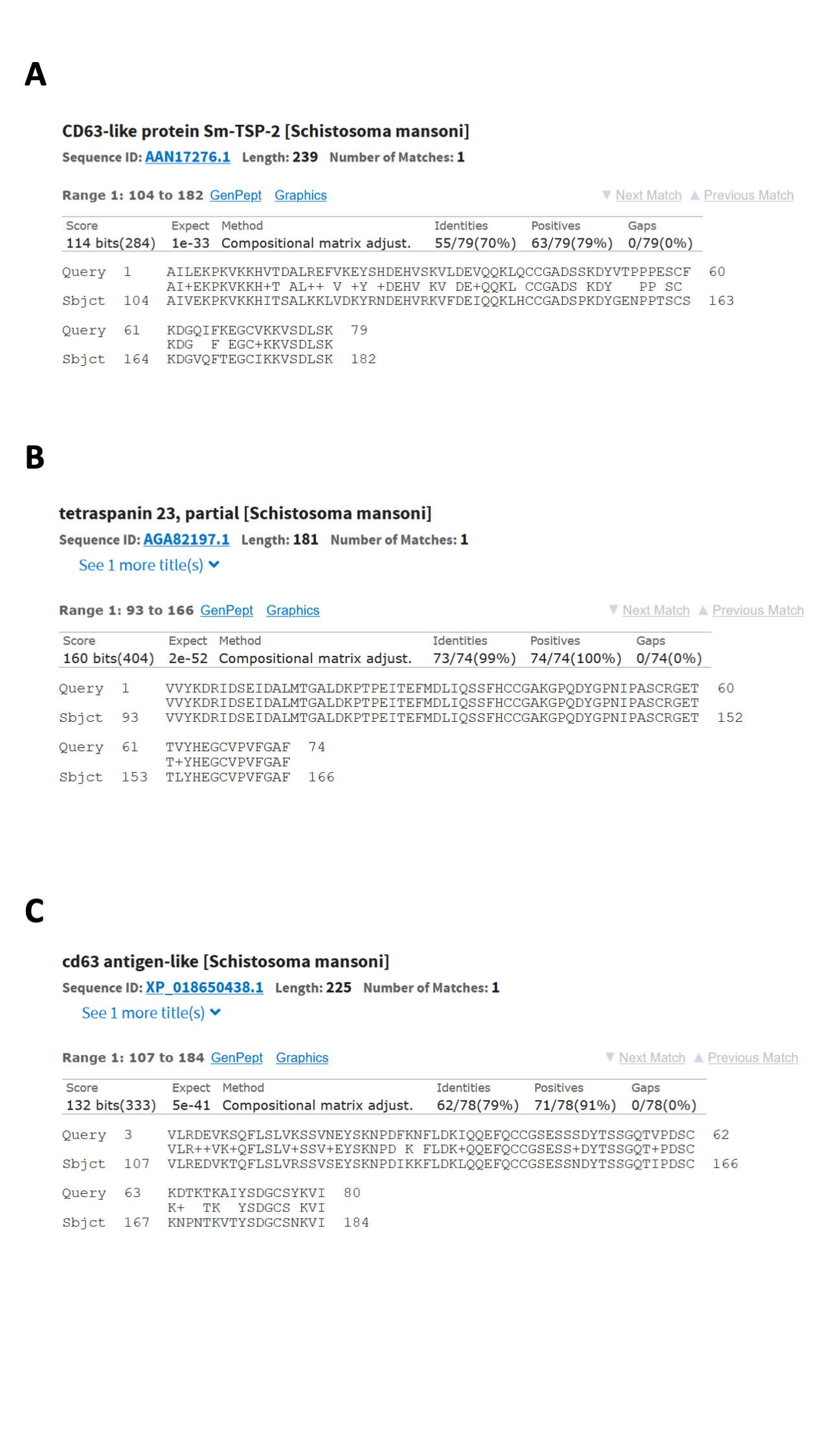

Supplement: Supplementary file 1 [file vaccines-08-00416-s001.zip › Figure_S1.png]
